# Construction of a multiple fluorescence labelling system for use in co-invasion studies of *Listeria monocytogenes*

**DOI:** 10.1186/1471-2180-6-86

**Published:** 2006-10-03

**Authors:** Jens B Andersen, Bent B Roldgaard, Ariel B Lindner, Bjarke B Christensen, Tine R Licht

**Affiliations:** 1 Danish Institute for Food and Veterinary Research, Department of Microbiological Food Safety, Mørkhøj Bygade 19, 2860 Søborg, Denmark; 2 Molecular, Evolution and Medical Genetics Laboratory, INSERM U571, Necker-Enfants Malades Faculty of Medicine, René Decartes – Paris V University, 156 Rue de Vaugirard, 75730 Paris Cedex 15, France

## Abstract

**Background:**

Existing virulence models are often difficult to apply for quantitative comparison of invasion potentials of *Listeria monocytogenes*. Well-to-well variation between cell-line based *in vitro *assays is practically unavoidable, and variation between individual animals is the cause of large deviations in the observed capacity for infection when animal models are used.

One way to circumvent this problem is to carry out virulence studies as competition assays between 2 or more strains. This, however, requires invasion-neutral markers that enable easy discrimination between the different strains.

**Results:**

A fluorescent marker system, allowing visualization and identification of single *L. monocytogenes *cells as well as colonies in a non-destructive manner, was developed. Five different fluorescent labels are available, and allowed simultaneous visual discrimination between three differently labelled strains at the single cell level by use of fluorescence microscopy. More than 90% of the *L. monocytogenes *host cells maintained the fluorescence tags for 40 generations.

The fluorescence tags did not alter the invasive capacity of the *L. monocytogenes *cells in a traditional Caco-2 cell invasion assay, and visual discrimination between invaded bacteria carrying different fluorescent labels inside the cells was possible.

**Conclusion:**

The constructed fluorescent marker system is stable, easy to use, does not affect the virulence of *L. monocytogenes *in Caco-2 cell assays, and allows discrimination between differently labelled bacteria after internalization in these cells.

## Background

*Listeria monocytogenes *is an intracellular pathogen of humans and animals [1], and has been implicated in several foodborne outbreaks as well as sporadic cases of disease during the last ten to fifteen years [2]. It is known that differences between strains as well as differences in environmental conditions affect the invasive potential of this pathogen [3-5]. Virulence models allowing comparison of the pathogenic potency of different cultures of *Listeria *are therefore essential tools in the study of bacteria within this genus. Additionally, such models are needed to study the role of specific genes in the process of infection.

Virulence models most commonly applied include cell-line based assays and various animal models. However, a number of obstacles are associated with the use of such models for comparative studies of the infectivity of *Listeria*. Despite efforts to optimize reproducibility in cell line based *in vitro *studies, well to well variation is difficult to avoid [6]. Likewise, in animal models, regardless of the employment of well-characterised laboratory animal strains, variations between individual animals commonly occur. The lack of accurate reproducibility in existing virulence models means that it is often difficult to compare the virulence of two different *Listeria *strains in a quantitative manner. A solution to this problem is to carry out virulence studies as competition assays between two or more strains. This approach has been applied with success by use of Listeria carrying resistance markers to various antibiotics [7]. An attractive alternative is to use fluorescent protein markers. The Green Fluorescent Protein (GFP) was introduced approximately ten years ago as a convenient tool for studies of the pathogenesis of e.g. *Salmonella typhimurium *and *Yersenia paratuberculosis *[8]. More recently, this protein has been applied in studies of Gram-positive bacteria including *Streptococcus suis *[9], and *Listeria monocytogenes *[10,11]. The advantage of fluorescent markers, when compared to resistance gene markers, is that they allow direct visualization and real-time studies of tagged bacteria. Furthermore, they allow certain identification of the tagged strains in un-sterile systems such as the mammalian gastrointestinal tract, where naturally occurring resistant bacteria may be difficult to distinguish from resistance-tagged strains. The disadvantage of fluorescence tagging is that small, tagged subpopulations can be difficult to quantify, since selection for fluorescence is not possible. This disadvantage may be overcome by combining fluorescent markers with antibiotic resistance markers.

We have developed a fluorescent marker system that allows identification of single *Listeria *cells as well as colonies in a non-destructive manner, and demonstrate its use in a traditional Caco-2 cell invasion assay.

## Results and discussion

### A multicolour tagging system based on the synthesis of Cyan Fluorescent Protein (CFP), Green Fluorescent Protein (GFP), Yellow Fluorescent Protein (YFP), DsRedExpress or HcRed

Fluorescent microscopy at 16× magnification revealed that *L. monocytogenes *ScottA harbouring either pNF8 (wt-GFP), pJEBAN2 (wt-CFP), pJEBAN3 (wt-YFP), pJEBAN4 (wt-HcRed) and pJEBAN6 (wt-DsRedEx) all gave rise to easily detectable fluorescent colonies of the expected colour, demonstrating that a heterolog expression of Green Fluorescent Protein (GFP), Cyan Fluorescent Protein (CFP), Yellow Fluorescent Protein (YFP), or the red fluorescent markers HcRed and DsRedexpress had successfully been established in *L. monocytogenes *ScottA. The labelling system was tested and shown to confer the expected fluorescence also to other strains of *L. monocytogenes*, including EGDe and LO28 (Table 1).

**Figure 1:**
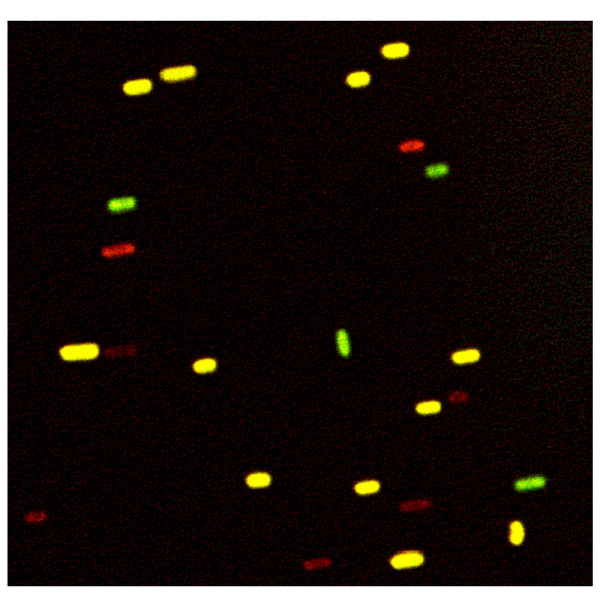
**Visualization of fluorescent *L. monocytogenes *cells**. Confocal image showing single cells of *L. monocytogenes *tagged with CFP, YFP or DsREdExpress. Over night cultures of each of the three strains were mixed in a 1:1:1 ratio prior to visualization. Exitation wavelengths were 456, 488 or 543 nm, and emission was captured at 500–510, 555–583 or 592–640 nm.

**Figure 2:**
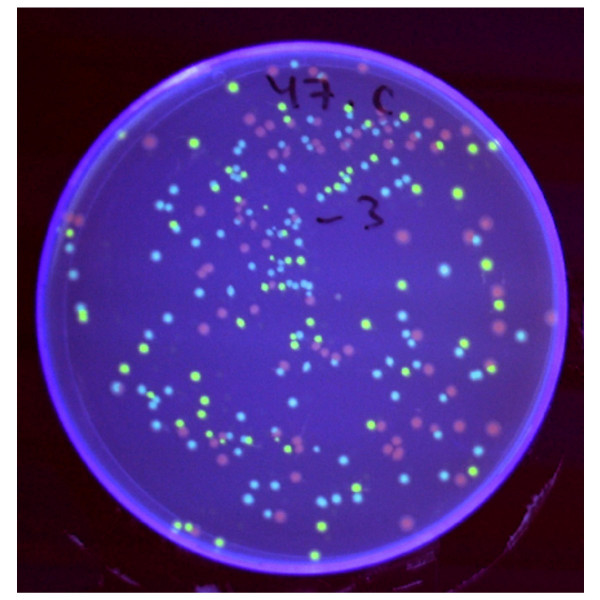
**Visualization of fluorescent *L. monocytogenes *colonies**. Colonies of wt-CFP, wt-YFP and wt-DsRedExpress could be discriminated when placed on a 302 nmUV-table, and appeared bluish, yellow and red.

*L. monocytogenes *cells tagged with any of the fluorescent proteins could be detected at the single cell level by fluorescence microscopy (630× magnification). By employment of appropriate combinations of different excitation wavelength and different filtersets, spectral discrimination between strains wt-CFP, wt-YFP and wt-DsRedEx mixed in a ratio of 1:1:1 was obtained at the single cell level (Figure 1). Although CFP, YFP and DsRedExpress are not maximally excitated by UV-light, visual inspection revealed that colonies of wt-CFP, wt-YFP and wt-DsRedEx could be discriminated when placed on a 302 nmUV-table (Figure 2). GFP tagged *Listeria *also gave rise to fluorescent colonies when excitated at 302 nm, however as expected from the similar spectral properties of GFP (emission maximum at 510 nm), and YFP (emission maximum at 527 nm), visual differentiation between GFP-tagged and YFP-tagged *L. monocytogenes *at the colony level was not possible. Similarly, visual discrimination between colonies of GFP- and CFP-tagged cells was difficult.

### Listerial growth and plasmid stability in batch cultures

No difference in growth rate between the labelled strains was observed during exponential growth in Erythromycin-supplemented BHI of *L. monocytogenes *ScottA tagged with plasmids carrying CFP, GFP, YFP, DsRedEx or HcRed. This observation is important for the utility of the tagged stains in comparative co-inoculation studies, where biases in growth could obscure the results.

**Figure 3:**
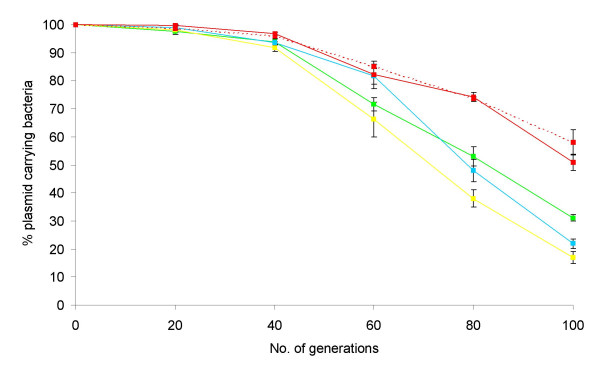
**Stability of fluorescence-encoding plasmids**. Segregational stability of plasmid pNT8 derivatives encoding the fluorescent proteins CFP (blue), GFP (green), YFP (yellow), DsRedExpress (red, solid line) or HcRed (red, broken line). *L. monocytogenes *harbouring the plasmids were propagated in BHI batch culture as described in the Methods section. Each point represents the average of data from three experiments. Error bars represent standard deviations.

Propagation of the five labelled strains for 100 generations without Erythromycin revealed that within the first 40 generations, more than 90 % of the bacteria in any of the five cultures still carried the plasmid. No significant differences in the fraction of plasmid harbouring cells were observed among the five cultures during this period, clearly demonstration that the segregational stability of pNF8, pJEBAN2, pJEBAN3, pJEBAN4 and pJEBAN6 was the same for all plasmids during the first 40 generations (Figure 3). Throughout the experiment, the fraction of plasmid harbouring bacteria as a function of the number of cell divisions was similar for cultures of *L. monocytogenes *tagged with CFP, GFP, and YFP, reaching a value of approximately 20 % following propagation for 100 generations in the absence of selective pressure. Likewise, the fraction of plasmid harbouring bacteria as a function of generation number was observed to be similar in cultures of HcRed- and DsRedEx-tagged cells, however more than 50% of these bacteria still carried the plasmid at the end of the experiment (Figure 3). This group of plasmids was thus slightly more stably maintained than the plasmids tagged with CFP, GFP and YFP, which are all derivatives of the same Green Fluorescent Protein isolated from the jellyfish *Aequorea victoria *[10,12]. The sequences of the red fluorescent proteins, which are obtained from the sea anemone *Heteractis crispa *[13] and the coral *Discosoma *[14], respectively, are shorter than the GFP-derived proteins, which may mean that they impose a smaller cell burden on the tagged *Listeria*, thus explaining the better stability. The observed differences in maintenance should be taken into consideration when applying the constructed plasmids (Table 1) for comparison experiments where fluorescence tagged bacteria are quantified after more than 40 bacterial generations.

Non-fluorescent, erythromycin resistant colonies were never observed in any of the 5 cultures investigated, confirming that all the constructed plasmids were 100 % structurally stable.

### Invasion assays with fluorescence-tagged bacteria

**Figure 4:**
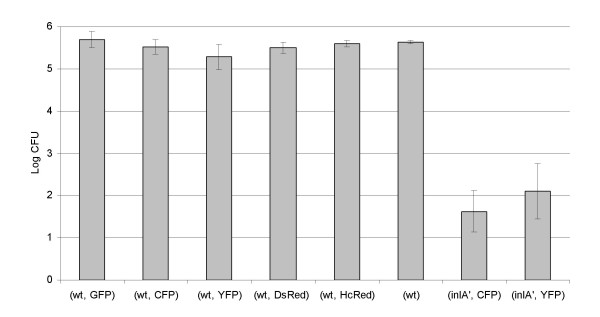
**Invasion potentials fluorescence tagged *L. monocytogenes***. Numbers of Colony Forming Units (CFU) of fluorescent *L. monocytogenes *strains and its inlA' mutant, which were internalized per well of Caco-2 cells. Counts were normalized to a concentration of 10^7 ^*L. monocytogenes *per ml in the bacterial cultures prior to infection of Caco-2 cells. Each point represents the average of data from two experiments. Error bars represent standard deviations. Statistical analysis (Dunnet's test) confirmed that the infection potentials of the labelled strains were not different from that of the wt (p < 0.05).

**Figure 5:**
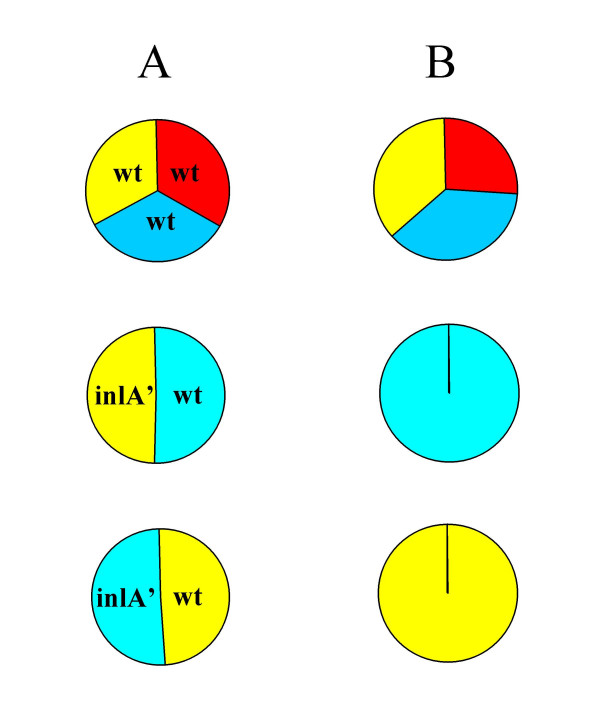
**Mixed infections**. Ratio of wt *L. monocytogenes *strains and its inlA' mutant tagged with YFP (yellow), DsRed (red) or CFP (blue) prior to invasion (A) and after recovery from Caco-2 cells (B).

Exposure of Caco-2 cells to the fluorescence tagged strains of *L. monocytogenes *ScottA at an infection multiplicity of approximately 25 bacteria per cell showed that infection potentials of these strains were not different (p < 0.05) from that of the un-tagged parent strain (Figure 4). Expression of the fluorescent proteins thus did not affect the infection potential of the investigated strain. Additionally, when wt strains with three different fluorescent tags were mixed in a ratio of 1:1:1 prior to invasion in Caco-2 cells, almost the same ratio was found between the tagged *Listeria *that had invaded the cells, showing that differently labelled strains were equally virulent (Figure 5). Caco-2 assays performed with the *L. monocytogenes *strains EGDe and LO28 tagged with either CFP or YFP showed that the invasion frequency was independent of these two fluorescent tags also in other strain backgrounds (data not shown).

It should be noted, that infection of mammalian cells does not give full information about infection at the levels of complex tissues and individuals. However, unpublished data from our lab indicated that also in a guinea-pig model, the virulence of *Listeria *was unaffected by the fluorescent labels [B. B. Roldgaard, J. B. Andersen, T.R. Licht and B.B. Christensen, unpublished]. This is in accordance with previous studies showing that GFP-tagging did not affect the virulence of *Listeria *in a murine model [10].

An *L. monocytogenes *ScottA strain carrying a mutation in the *inlA *gene, encoding InternalinA which is known to be necessary for listerial epithelial invasion [15] was constructed and designated inlA'. The infection potential of the inlA' mutant was more than 1000 fold lower than observed for the wt strains (Figure 4), which is in accordance with previous reports [16,17].

When fluorescence-tagged *L. monocytogenes *wt strains were mixed with the fluorescence-tagged invasion-impaired inlA' mutant, the difference in invasion capacity observed for the individual strains was reflected also in the mixed infections (Figure 5). However, since it was not possible to select for the mutant strain, the detection limit was increased by the presence of the wt strain, which outnumbered the mutant about 1000 fold as expected from the separate invasion assays (Figure 4).

**Figure 6:**
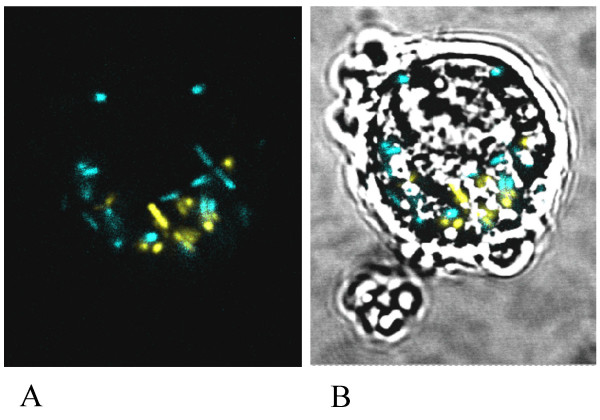
**Visualization of internalized bacteria**. Caco-2 cell containing a mixture of blue/cyan wt-CFP and yellow wt-YFP *L. monocytogenes*. A: Fluorescence microscopy image. B: Overlay of fluorescent image and phase contrast image showing the Caco-2 cell.

This demonstrates that co-invasion assays may be a useful method for rapid screening of differences in infection potentials between different strains of *L. monocytogenes *when a fluorescent labelling system is used for discrimination between the strains. Additionally, it shows that the presence of the wt strain does not mediate passive uptake of the inlA' mutant. Fluorescent bacteria were easily visualized and distinguished from each other inside the Caco-2 cells by fluorescence microscopy (Figure 6).

## Conclusion

We have developed a stable fluorescent labelling system for use in *L. monocytogenes*, and demonstrate that the labels do not affect the invasion potential of *L. monocytogenes *ScottA nor of an invasion-impaired mutant of this strain. The fluorescent labels allows easy discrimination between tagged cells and other bacteria in un-sterile systems, as well as discrimination between strains carrying different fluorescent markers, and provides a highly useful tool for researchers addressing virulence mechanisms of *Listeria*. Additionally, the labelling system allows real-time studies and visualization of bacteria internalized in eukaryotic cells, and is an optimal tool for comparative co-infection studies between up to three *Listeria *strains. However, for comparison of strains with highly different infection potentials, such as a wt *Listeria *and its *inlA *mutant, it should be taken into account that it is not possible to select for fluorescence, and that visualization of a strain constituting a very small minority might therefore be difficult. One solution to this would be to equip the less virulent strain with a distinct antibiotic resistance.

## Methods

### Bacterial strains, plasmids and growth media

Strains and plasmids used in this study are listed in Table 1. Bacterial strains were grown on BHI-agar supplemented with the relevant antibiotics. Erythromycin was used at a final concentration of 5 to 10 μg/mL for *L. monocytogenes *and of 150 ug/mL for *E. coli*. Nalidixic acid was used at a final concentration of 100 μg/mL for *L. monocytogenes*.

### DNA manipulation

Standard techniques for DNA manipulation were applied [18]. Plasmid DNA was prepared using the QIAGEN mini spin prep Kit (QIAGEN, Germany) and chromosomal DNA was extracted by the QIAGEN Genomic DNA Tip 100/G System (QIAGEN, Germany). Transformation of *E. coli *and *L. monocytogenes *was done by electroporation in a 0.2 cm cuvette by use of a GenePulser apparatus (Bio-Rad) set to 25 μF, 400 Ω and 2.5 kV/cm. PCR was carried out using the Phusion High Fidelity PCR system (FINNZYMES OY, Finland). A GFX PCR DNA and Gel Band Purification Kit (Amersham Pharmacia Biotech) were applied for purification of DNA fragments.

### Construction of a multi colour tagging system

Optimized version of the Cyan- and Yellow fluorescent proteins were created as follows: The hsp60 promoter driven GFP+ [19] was amplified and cloned into the pUC18 plasmid. pUC-yfp^++ ^was obtained by introducing the following mutations by site-directed mutagenesis (Stratagene): F46L, S65G, V68A, S72A, S175G, T203Y, whereas the pUC-cfp^++^, was obtained by introducing the K26R, Y66W, N146I, N164H, S175G, N212K mutations. Sequences can be found at [20]. The resulting proteins exhibit improved stability at elevated temperature (up to 46°C) and stronger fluorescence as judged by comparison with commercially available variants.

To construct a multicolour tagging system, the *cfp*^++ ^gene of pUC-cfp^++^, the *yfp*^++ ^gene of pUC-yfp^++^, the *HcRed *gene of pHcRed1 and the *DsRed-Express *gene of pDsRed-Express (Clontech Laboratories Inc., USA), were initially PCR amplified using either of the primersets Gfp_for/Gfp_rev, HcRed_for/HcRed_rev, or RedEx_for/DsRedEx_rev (Table 1). The four resulting PCR products encoding *cfp*^++^, *yfp*^++^, *HcRed *and *DsRed-Express *respectively, were restricted with BamHI and PstI and subsequently ligated to the 7 Kb BamHI-PstI fragment of pNF8, to give pJEBAN2, pJEBAN3, pJEBAN4 and pJEBAN6. Finally, pJEBAN2, pJEBAN3, pJEBAN4, pJEBAN6 and pNF8 were electroporated into *E. coli *strain S17-λ-pir to obtain donor strains from which pJEBAN2, pJEBAN3, pJEBAN4, pJEBAN6 and pNF8 can be conjugated into a broad range of bacteria by a simple 2 strain mating procedure.

### Fluorescent tagging of *L. monocytogenes*

All matings were carried out using a slightly modified version of the protocol previously described by Trieu-Cout and coworkers [21]. Briefly, 250 uL culture of *L. monocytogenes *ScottA (recipient) cultivated for 18 hours at 37°C in BHI broth containing 100 μg/mL Nalidixic acid and 250 μL culture of S17-λ-pir harbouring pJEBAN2 (donor) cultivated for 18 hours at 37°C in BHI broth containing 150 μg/mL erythromycin was harvested by centrifugation, washed twice with 250 μL of 0.9 % NaCl to remove antibiotics and subsequently resuspended in 25 μL of 0.9 % NaCl. The 25 μL aliquots of the donor and the recipient were mixed and subsequently spotted onto a BHI-agar plate containing 0.5 μg/mL penicillinG. Following 24 hours of incubation at 37°C, the resulting conjugation spots were gently removed from the BHI-agar plate and resuspended in 1 mL 0.9 % NaCl. Finally, in order to select Cyan fluorescent transconjugants (strains harbouring pJEBAN2), appropiate dilutions of the conjugation mix were spread onto BHI-agar containing 100 μg/mL nalidixic acid and 10 μg/mL erythromycin. By analogy, pNF8, pJEBAN3, pJEBAN4 and pJEBAN6 were conjugated from S17-λ-pir into *L. monocytogenes *to give green fluorescent, yellow fluorescent and red fluorescent strains. The inlA' mutant of *L. monocytogenes *(construction described below) was tagged with pJEBAN2 and pJEBAN3 in a similar way.

### Plasmid stability *in vitro*

To obtain starter cultures in which the entire *L. monocytogenes *populations carried pNF8, pJEBAN2, pJEBAN3, pJEBAN4 or pJEBAN6, brightly fluorescent single colonies of the strain harbouring either of these plasmids were initially inoculated into 10 mL BHI supplemented with 100 μg/mL Nalidixic acid and 10 μg/mL Erythromycin and incubated at 37°C for 18 hours. At timepoint g = 0 (number of population generations in the absence of selective pressure), 10 μl of each of the starter cultures were inoculated into 10 mL BHI supplemented with 100 μg/mL of Nalidixic acid (non selective media) and from the resulting cultures, samples were immediately taken to determine the fraction of plasmid harbouring bacteria at the start of the experiment (g = 0). Following 12 hours of growth in the absence of selective pressure (time point g = 10), 10 uL of each of the outgrown cultures were again inoculated into 10 mL of fresh medium, and from the resulting cultures samples were taken to determine the fraction of fluorescent bacteria (plasmid harbouring bacteria) at timepoint g = 10. This procedure was repeated every 12 hours for a total of 120 hours equivalent to approximately 100 generations. Following 24 hours of incubation at 37°C, all BHI-agar plates were replicated to BHI-agar plates supplemented with 10 μg/ml of Erythromycin, and the fraction of plasmid harbouring bacteria was determined as the number of Erythromycin resistant colonies divided by the total number of colonies present on the BHI-agar master plate. It was verified that all Erythromycin-resistant colonies were fluorescent by inspection on a UV-table (exitation 302 nm). All sampling was done in duplicates. The experiment was repeated 3 times.

### Construction of an *L. monocytogenes *deletion mutant designated inlA'

A mutant of *L. monocytogenes *ScottA carrying an in-frame deletion of the last 771 bp of the *inlA *gene was constructed using 2-way splicing by overlap extension (SOE) PCR [22] followed by allelic exchange mutagenesis. Initially, 2 PCR fragments designated InlA-int (293 bp) and InlA-down (270 bp) covering different regions of the *inlA *gene was amplified from chromosomal DNA of *L. monocytogenes *by use of the primersets, InlA_N_up/InlA_N_down and InlA_C_up/InlA_C_down, respectively (Table 2). Since the *inlA *gene of *L. monocytogenes *ScottA had only been partially sequenced at the time of design, primers were designed based on the genome sequence of *L. monocytogenes *4b 2365 [23]. In a second round of PCR, fragments InlA-int and InlA-down were spliced together by use of the primerset InlA_N_up/InlA_C_down to produce a 538 bp fragment (designated InlA') covering nt 1381 to nt 1630 directly followed by nt 2401 to nt 2652 of *inlA*. The PCR fragment InlA' was restricted with BamHI and HindIII, and cloned in between the BamHI and HindIII sites of the temperature sensitive shuttle vector pAUL-A [24]. The resulting plasmid pJEBAN12 was introduced into *L. monocytogenes *by electroporation and *inlA *mutants carrying inframe deletions covering the last 771 nt of the *inlA *gene were subsequently constructed as previously described [25]. Finally, strains carrying the desired deletion were identified by PCR, and the correct deletion event was verified by DNA sequence analysis of the resulting PCR product.

### Caco-2 invasion assays

Enterocyte-like Caco-2 cells were cultivated and prepared as previously described [3]. Bacteria were cultivated over night at 37°C in BHI media supplemented with 10 μg/mL of Erythromycin and diluted to a concentration of 10^7 ^bacteria/mL prior to the invasion assays. For co-invasion assays, the two or three strains were mixed in a 1:1 or 1:1:1 ratio. One ml of bacterial culture was applied to each well, resulting in a multiplicity of infection of approximately 25 bacteria per Caco-2 cell. Following 1 hour of invasion and 2 hours of gentamycin treatment to kill extracellularly located bacteria, Caco-2 cells were lysed, diluted and subsequently spread onto BHI-agar supplemented with 10 μg/mL of Erythromycin. After 96 hours of incubation at 22°C, plates were placed on a UV table (excitation at 302 nm), fluorescent colonies were enumerated, and digital images were recorded as described below. Sampling was done in triplicate, and the experiments were performed twice.

### Fluorescence microscopy

Fluorescence microscopy was carried out by use of a Leitz DM RBE microscope fitted with filter cubes G/R, I3 and N2.1 (Leica Microsystems Heidelberg GmbH, Germany).

Microscopic observations and image acquisitions were performed with a model TCS SP1 three channel scanning confocal laser microscope (Leica Microsystems Heidelberg GmbH, Germany) equipped with an argon laser (458 nm, 476 nm, 488 nm and 514 nm wavelength) and two HeNe lasers (543 nm and 633 nm wavelength) and a variable spectrophometric detection system. The Leica Confocal Sofware TCS SP/NT version 2.5.1347 was used.

## Authors' contributions

JBA and ABL carried out the molecular constructions. JBA carried out the invasion assays and BBR did the microscopy. BBC and TRL conceived of the study and participated in its design and coordination. JBA and TRL drafted the manuscript. All authors red and approved the final manuscript.

## Figures and Tables

**Table 1:** Strains and plasmids

Strains	Relevant genotype and characteristics	Reference
*E. coli*.		
S17-1 λ pir	*thi rspL *(Str ^r^) *endA sbcB15 sbcC hsdR *(r_k_^- ^m_k_^-^) Δ (*lac-proAB*) [F' *traD36 lacI*^q ^Δ(*lacZ*)*M15 proA*^+^*B*^+^]	[26]
JM105	Cloning host. *thi rspL *(Str ^r^) *endA sbcB15 sbcC hsdR *(r_k_^- ^m_k_^-^) Δ(*lac-proAB*) [F' *traD36 lacI*^q ^Δ (*lacZ*)*M15 proA*^+^*B*^+^]	[27]
*L. monocytogenes *EGDe	Host strain	[28]
*L. monocytogenes *LO28	Host strain	[29]
*L. monocytogenes *ScottA	Host strain	[30]
Wt	Nal^R ^mutant of *L. monocytogenes *ScottA (wt)	
inlA'	Nal^R^; *inlA *mutant of *L. monocytogenes *ScottA (wt) carrying an inframe deletion of the last 800 bp of the inlA gene.	This study
wt-CFP	Nal^R^; Em^R^; (ScottA wt) harbouring pJEBAN2	This study
wt-GFP	Nal^R^; Em^R^; (wt) harbouring pNF8	This study
wt-YFP	Nal^R^; Em^R^; (ScottA wt) harbouring pJEBAN3	This study
wt-HcRed	Nal^R^; Em^R^; (ScottA wt) harbouring pJEBAN4	This study
wt-DsRed	Nal^R^; Em^R^; (ScottA wt) harbouring pJEBAN6	This study
inlA'-CFP	Nal^R^; Em^R^; (ScottA *inlA'*) harbouring pJEBAN2	This study
inlA'-YFP	Nal^R^; Em^R^; (ScottA *inlA'*) harbouring pJEBAN3	This study

Plasmids		

pUC-cfp^++^	Ap^R^; donor of *cfp*^++ ^gene	This study
pUC-yfp^++^	Ap^R^; donor of *yfp*^++ ^gene	This study
PhcRed1	Ap^R^; donor of *HcRed *gene	Clontech laboratories Inc.
PdsRed-Express	Ap^R^; donor of *DsRed-Express *gene	Clontech laboratories Inc.
pNF8	Em^R^, Mob^+^(IncP), *oriR *pAMβ1, *oriR *pUC; *Pdlt-gfpmut1*	[10]
PJEBAN2	Em^R^, Mob^+^(IncP), *oriR *pAMβ1, *oriR *pUC; *Pdlt-cfp+*	This study
PJEBAN3	Em^R^, Mob^+^(IncP), *oriR *pAMβ1, *oriR *pUC; *Pdlt-yfp+*	This study
PJEBAN4	Em^R^, Mob^+^(IncP), *oriR *pAMβ1, *oriR *pUC; *Pdlt-HcRed*	This study
PJEBAN6	Em^R^, Mob^+^(IncP), *oriR *pAMβ1, *oriR *pUC; *Pdlt-DsRedExpress*	This study
pAUL-A	Em^R^,	[24]
pJEBAN12	Em^R^, pAUL-A carrying a 538 bp PCR product that give rise to an inframe deletion of the last 771 bp of *InlA *when recombined into the chromosome of *L. monocytogenes *ScottA	This study

**Table 2:** Primers designed in this study.

Primer	Sequence
Gfp_for	5'-ATATAGGATCCGAAAGGAGGTTTATTAAAATGGCTAGCAAAGGAGAAGAACTTT-3' *BamHI*
Gfp_rev	5'-TTTTTCTGCAGTTATTTGTAGAGCTCATCCATGCC-3' *PstI*
HcRed_for	5'-ATATAGGATCCGAAAGGAGGTTTATTAAAATGGTGAGCGGCCTGCTGAAGG-3' *BamHI*
HcRed_rev	5'-TTTTTCTGCAGTTAGTTGGCCTTCTCGGGCAGGTC-3' *PstI*
DsRedEx_for	5'-ATATAGGATCCGAAAGGAGGTTTATTAAAATGGCCTCCTCCGAGGACGTC-3' *BamHI*
DsRedEx_rev	5'-TTTTTCTGCAGTTACAGGAACAGGTGGTGGSGG-3' *PstI*
InlA_N_up	5'-ATATAGGATCCGCGGCCGCGAATTTACCTAGTTATACCAATGAAGT-3' *BamHI NotI*
InlA_N_down	5'-CATCTAGCTCTTTACACTACTTCTAAGTTCCGCCGGTTTGGGCATC-3'
InlA_C_up	5'-TAGAAGTAGTGTAAAGAGCTAGATG-3'
InlA_C_down	5'-ATATAAAGCTTGCGGCCGCTTCTGCAAAAGCATCATCTGGAAAA-3' *HindIII NotI*

Underlined areas mark targets for the indicated restriction enzymes.
